# The Nitric Oxide Donor Pentaerythritol Tetranitrate Reduces Platelet Activation in Congestive Heart Failure

**DOI:** 10.1371/journal.pone.0123621

**Published:** 2015-04-30

**Authors:** Ulrike Flierl, Daniela Fraccarollo, Julian D. Widder, Jan Micka, Jonas Neuser, Johann Bauersachs, Andreas Schäfer

**Affiliations:** Klinik für Kardiologie und Angiologie, Medizinische Hochschule Hannover, Germany; University Hospital Medical Centre, GERMANY

## Abstract

**Background:**

Platelet activation associated with endothelial dysfunction and impaired endogenous platelet inhibition is part of the cardiovascular phenotype of congestive heart failure (CHF) and contributes to the increased risk for thromboembolic complications. Pentaerythritol tetranitrate (PETN) has been shown to release nitric oxide without development of nitrate tolerance. We investigated the effect of chronic PETN treatment on platelet activation and aggregation in an experimental CHF model.

**Methods and Results:**

Chronic ischemic heart failure was induced in male Wistar rats by coronary artery ligation. Starting 7 days thereafter, rats were randomised to placebo or PETN (80 mg/kg twice daily). After 9 weeks, activation of circulating platelets was determined measuring platelet bound fibrinogen, which requires activated glycoprotein IIb/IIIa on the platelet surface. Binding was quantified by flow-cytometry using a FITC-labelled anti-fibrinogen antibody. Platelet-bound fibrinogen was significantly increased in CHF-Placebo (mean fluorescence intensity: Sham 88±4, CHF-Placebo 104±6, p<0.05) and reduced following treatment with PETN (89±7, p<0.05 vs. CHF-Placebo). Maximal and final ADP-induced aggregation was significantly enhanced in CHF-Placebo vs. Sham-operated animals and normalized / decreased following chronic PETN treatment. Moreover, platelet adhesion was significantly reduced (number of adherent platelets: control: 85.6±5.5, PETN: 40±3.3; p<0.001) and VASP phosphorylation significantly enhanced following *in vitro* PETN treatment.

**Conclusion:**

Chronic NO supplementation using PETN reduces platelet activation in CHF rats. Thus, PETN may constitute a useful approach to prevent thromboembolic complications in CHF.

## Introduction

Endothelial dysfunction is a common phenomenon occurring in various diseases such as diabetes, coronary artery disease (CAD) [1] and congestive heart failure (CHF) [2]. The presence of endothelial dysfunction is associated with an increased mortality risk in patients with chronic heart failure [3]. Moreover, it is well acknowledged that patients with CHF are at risk of suffering from thromboembolic complications [4]. Amongst other procoagulatory factors, activated platelets are important contributors to a thromboembolic environment in heart failure [5] possibly contributing to cerebral and peripheral thromboembolic events [6]. In turn, platelet activation is influenced by impaired endogenous inhibition mechanisms, such as lack of generation of nitric oxide (NO). NO is the most important autacoid being indispensable for maintaining endothelial integrity and controlling vascular tone. One major source of endogenous NO are endothelial cells which generate NO via endothelial NO synthase (eNOS) [7]. Reduced NO bioactivity and increased formation of reactive oxygen species (ROS) results in impaired coronary and systemic perfusion and reduced exercise capacity of CHF patients. Besides its essential endothelial actions, NO is also crucial for the regulation of platelet activation, adhesion and aggregation [8]. Hence, inhibition of endogenous NO supply results in rapid platelet activation *in vivo* [9]. Moreover, in the setting of an experimental animal model of CHF, platelet activation could be attenuated by enhancing systemic NO bioavailability [10]. One option how to exogenously apply NO is the use of organic nitrates causing vasodilation by the induction of NO formation [11].

Undoubtedly, nitrate treatment has a top-ranking in acute relief of anginal chest pain [12]. However, chronic use of most organic nitrates results in nitrate tolerance and aggravation of endothelial dysfunction. The mechanism behind these phenomena is still under investigation. Presumably, counter-regulatory mechanisms are induced upon long term use such as vasoconstriction by increased levels of vasoconstrictors (e.g. angiotensin II, noradrenalin) and generation of oxidative stress [13]. To which extent platelets are affected by nitrate induced adverse effects is unclear [14, 15]. As compared to other organic nitrates such as nitroglycerin (GTN), isosorbide monohydrate (ISMN) and isosorbide dihydrate (ISDN), the long acting drug pentaerythritol tetranitrate (PETN) had been shown to release NO without the development of afore mentioned unfavourable side effects [16]. Quite the contrary, PETN was found to actively improve endothelial function by induction of the anti-oxidant enzyme heme oxygenase-1 (HO-1) [17]. So far, clinical trials where PETN was evaluated in CAD [18] and angina pectoris patients [19] rather disappointingly showed no overall additional benefit regarding anti-ischaemic efficacy or overall endothelial function. However, some improvement in exercise tolerance in selected patient subgroups and positive changes in microcirculation were present.

In healthy dogs, PETN administration did not adversely affect platelet function, [20], the effects of chronic PETN treatment on platelet function in disease models are unknown.

Therefore, we investigated the effects of chronic PETN treatment on platelet activation and aggregation in an experimental CHF model.

## Methods

Experimental procedures on animals met the requirements of the German legislation on protection of animals and were approved by the Government of Bavaria (permit number 54–2531.01-48/07), Germany, in accordance with EU Directive 2010/63/EU. All surgery (coronary ligation or Sham operation as well as haemodynamic measurements and sacrifice) was performed under deep isoflurane anaesthesia determined by total absence of reaction to pain under spontaneous respiration, and all efforts were made to minimize suffering.

### Human blood samples

For *in vitro* experiments, blood samples were collected from healthy donors who gave written informed consent and who had not taken any medication within the last 10 days. The collection of blood samples for in vitro research had been approved by the ethics committee at the University of Würzburg (permit number 165/06).

### Animals and induction of CHF

Sham-operation or left coronary artery ligation was performed in adult male Wistar rats (250–300 g, obtained from Harlan-Winkelmann, Borchen, Germany) as previously described [21]. Rats were then randomised to placebo or PETN (80 mg/kg twice daily given by gavage). After 10 weeks hemodynamic studies were performed to ensure that the animals were in the chronic stable phase of heart failure [21]. CHF was defined by elevated left ventricular end-diastolic pressure (LVEDP, >15 mmHg) and impaired left ventricular function [10]. Moreover, platelet activation was assessed for experiments under chronic treatment at least 12 hours after the last dose of PETN had been administered.

### Sample collection and determination of infarct size

After hemodynamic measurement, the heart was removed and the left ventricle was then cut into three transverse sections: apex, middle ring (3 mm), and base. From the middle ring, 5 μm-sections were cut at 100 μm-intervals and stained with picrosirius red. The boundary length of the infarcted and non-infarcted surfaces of the endocardium and the epicardium were traced with a planimeter digital image analyser and infarct size (fraction of the infarcted left ventricle) was expressed as a percentage of length and only rats with extensive infarcts (>45%) meeting the above mentioned haemodynamic criteria were included in the platelet reactivity studies.

### Platelet sampling

Blood samples were taken from the right carotid artery into tubes containing 3.8% sodium citrate before hemodynamic measurements. General anesthesia was induced using isoflurane, sufficiency of anesthesia was determined by total absence of reaction to pain under spontaneous respiration.

### Flow cytometry

Whole blood was diluted with PBS (free of Ca^2+^ and Mg^2+^, enriched with D-Glucose [5.5 mmol/L] and 0.5% BSA). Platelet bound fibrinogen, which requires activated glycoprotein IIb/IIIa on the platelet surface, was determined by incubation with a FITC-labeled anti-fibrinogen antibody (WAK-Chemie, Steinbach, Germany).

Following incubation with the antibody, platelets were fixed with methanol-free formaldehyde (1.5%) for 10 min, and subsequently analyzed in a Becton Dickinson FACSCalibur at a low flow rate. The platelet population was identified on its forward and side scatter distribution, and 20,000 events were analyzed for mean fluorescence using CELLQuest software, version 3.1f. Unspecific binding was arbitrarily adjusted to a mean fluorescence of 10.

### Platelet aggregation

Platelet aggregation was analyzed using a platelet aggregation profiler (PAP-8, MöLab, Hilden, Germany). Citrated whole blood was centrifuged at 180 g for 10 min to obtain platelet-rich plasma (PRP), which was diluted with PBS to obtain a final platelet concentration of 250.000/μL. ADP in different concentrations (1/2/5/10/20μM) was used to induce aggregation.

### Platelet adhesion

Circular glass cover slips were incubated with fibrinogen (30 μg/mL) at 4°C overnight. After washing with modified Tyrode buffer, the cover slips were blocked with 1% BSA. PRP was pre-incubated with PETN (5μg/mL) or the solvent DMSO for 30 minutes at room temperature. Then, platelets were allowed to adhere on the coated cover slips for 30 minutes at 37°C, washed with modified Tyrode buffer, permeabilized and fixed with 1%Triton X-100 and CellFix (Becton Dickinson). To visualize the actin cytosceleton and focal adhesions in microscopy, platelets were stained with an TRITC-conjugated phalloidin and anti-Vinculin staining for 30 minutes, then washed and stained with a FITC-labeled goat anti-mouse antibody. For staining of Vasodilator-stimulated phosphoprotein (*VASP*), adherent platelets were incubated with FITC-conjugated phospho-Ser 239 antibody (nanoTools) or isotype control antibody, respectively. Finally, cover slips were mounted using Vectashield (Vector) and images were taken on an Olympus BX61 microscope with a 60x and 100x magnification.

### Substances

Unless stated otherwise, all chemicals were obtained from Sigma (Deisenhofen, Germany) in the highest purity available. PETN was kindly provided by actavis (Munich, Germany).

### Statistics

Data are presented as means ± SEM and analyzed using Student’s t-test or one-way ANOVA with a Tukey post-hoc test where appropriate. A repeated measures ANOVA was applied for dose response curves. A p<0.05 was considered statistically significant.

## Results

Hemodynamics were significantly different between sham-operated and CHF groups, illustrating the existence of congestive heart failure. There were no significant differences in terms of systemic hemodynamics between placebo- and PETN-treated CHF groups when the experiments were performed in the absence of an acute drug effect (Table 1).

**Table 1:** Hemodynamics and descriptive parameters from placebo- and PETN-treated CHF rats compared with sham-operated placebo-treated rats.

	Sham	CHF Placebo	CHF PETN
***N***	12	11	10
**MI (%)**	-	53 ± 1	51±1
**LV (mg)**	807 ± 21	832 ± 23	848±10
**RV (mg)**	146 ± 7	368 ± 24*	350±35*
**BW (g)**	424.8 ± 18.7	406.3 ± 10	421.1±12
**LVSP (mmHg)**	159.9 ± 7	120.6 ± 5*	121.6±5*
**LVEDP (mmHg)**	4.6 ± 0.4	29.2 ± 3.3*	28.2±4.1*
leukocytes (n*1000/μ**l)**	5.97 ± 0.57	7.56 ± 0.47	8.30±0.65
monocytes (n*1000/μ**l)**	0.26 ± 0.03	0.41 ± 0.05*	0.25±0.04^#^
platelets (n*1000/μ**l)**	809 ± 20	774 ± 22	764±40
**MPV (fl)**	6.45 ± 0.07	6.45 ± 0.07	6.59±0.12

CHF was defined by elevated left ventricular end-diastolic pressure (LVEDP, >15 mmHg) and impaired left ventricular function, values are means ± standard error (* = p<0.01 vs. Sham;^#^ = p<0.05 vs. CHF Placebo). MI = myocardial infarction; LV = left ventricle; RV = right ventricle; BW = body weight; LVSP = left-ventricular systolic pressure; LVEDP = left-ventricular end-diastolic pressure; MPV = mean platelet volume

### Fibrinogen-binding as marker of in vivo platelet activation

**Fig 1:**
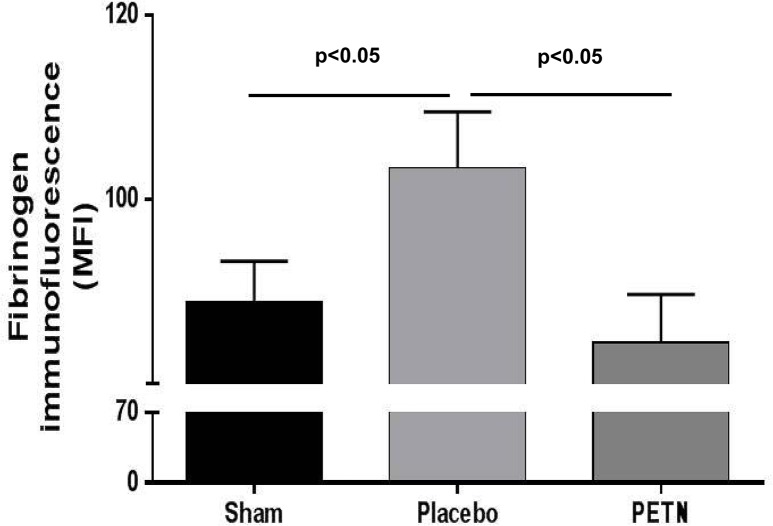
Activation of circulating platelets in CHF. Platelet-bound fibrinogen is significantly increased in CHF-placebo (mean fluorescence intensity (MFI): Sham 88±4, CHF-placebo 104±6, p<0.05) and reduced following treatment with PETN (89±7, p<0.05 vs. CHF-placebo), n = 8–12.

The extent of in vivo platelet activation was measured by analysis of platelet-bound fibrinogen reflecting glycoprotein IIb/IIIa activation in unstimulated whole blood. Platelet-bound fibrinogen was significantly elevated in CHF compared to placebo (MFI: sham 88±4, CHF-placebo 104±6, p<0.05) confirming the existence of platelet activation in the setting of heart failure. PETN treatment reduced excessive fibrinogen-binding in CHF-animals to the level of sham-operated rats (MFI: 89±6, p<0.05 vs. CHF-placebo) (Fig 1).

### Effects of chronic PETN treatment on platelet aggregation

**Fig 2:**
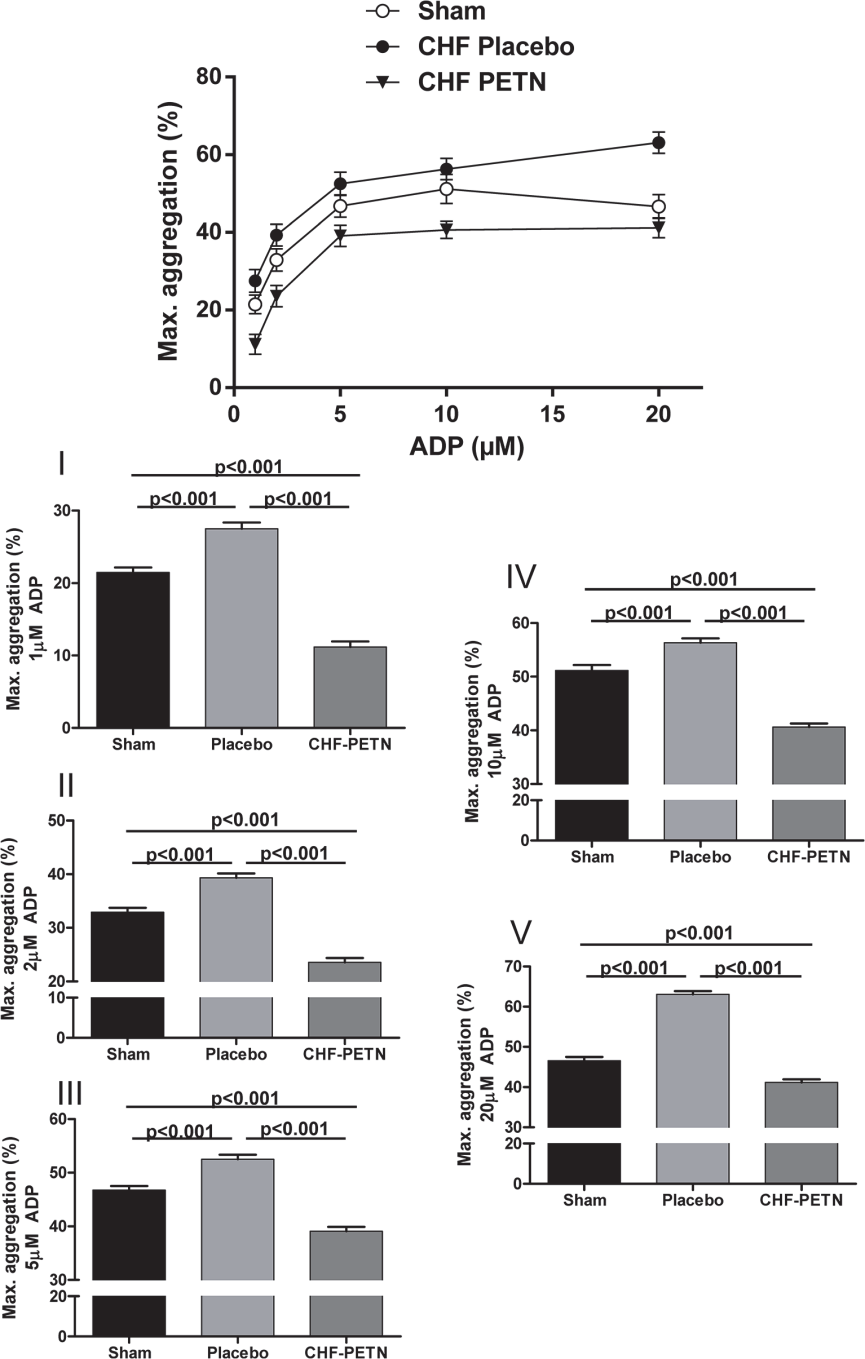
Maximal aggregation of platelets from CHF rats ex vivo. Maximal ADP-induced aggregation was significantly enhanced in CHF-placebo vs. sham-operated animals at different ADP concentrations and normalized and partly even decreased following chronic PETN treatment, n = 10–12; **I-V** = different ADP concentrations in detail (1/2/5/10/20μM ADP).

**Fig 3:**
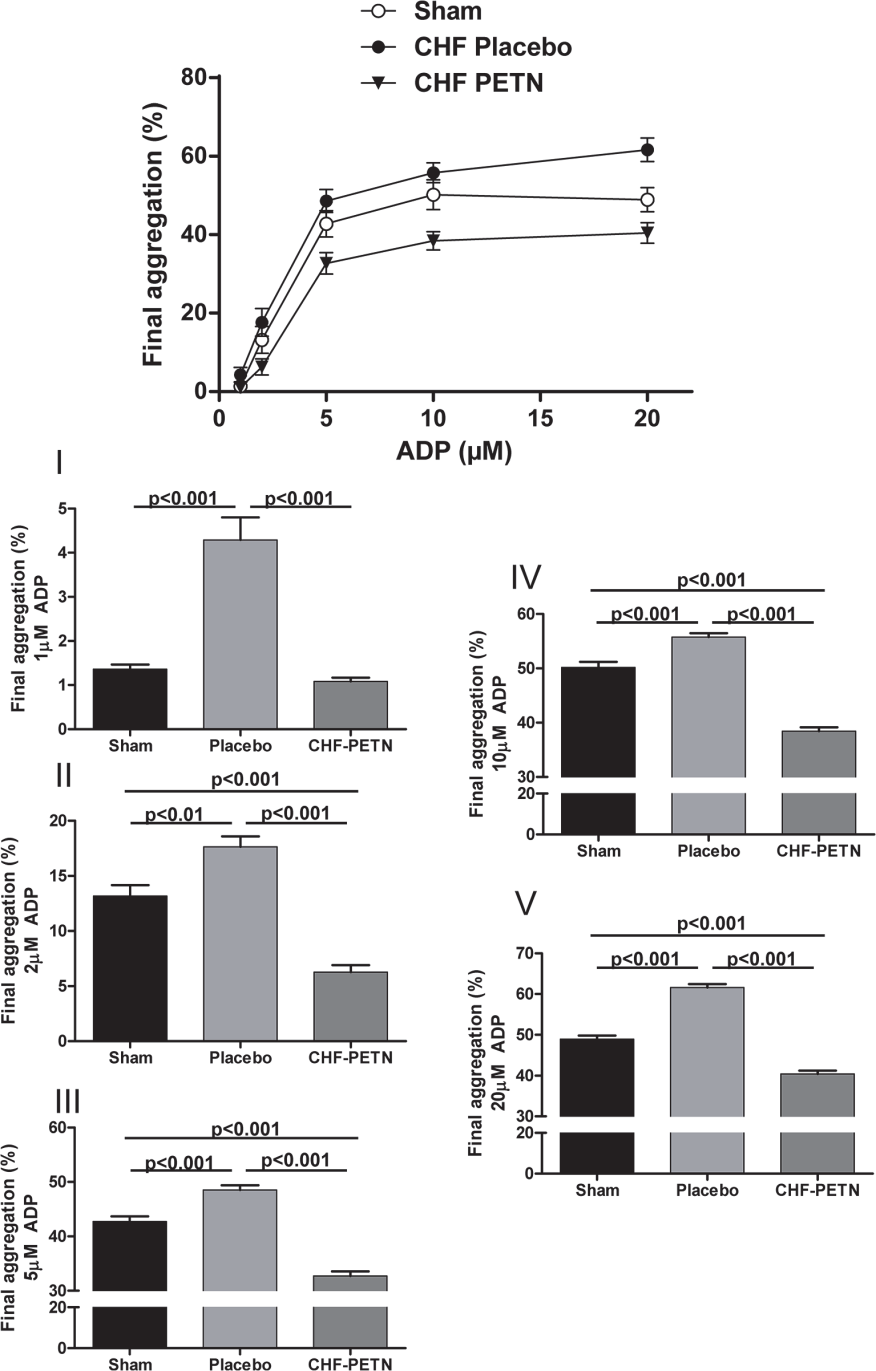
Final aggregation of platelets from CHF rats ex vivo. Final ADP-induced aggregation was significantly enhanced in CHF-placebo vs. sham-operated animals at different ADP concentrations and normalized and partly even decreased following chronic PETN treatment, n = 10–12; **I-V** = different ADP concentrations in detail (1/2/5/10/20μM ADP).

ADP induced platelet aggregation was significantly enhanced in platelet-rich plasma from CHF vs. sham-operated rats for various ADP concentrations (1/5/10/20μM) affecting maximal (Fig 2) as well as final (Fig 3) aggregation. The increase in platelet reactivity due to heart failure was attenuated following chronic treatment with the long-acting nitrate PETN in CHF-animals. On top of a significant decrease of platelet aggregation levels, aggregation observed for samples from PETN treated CHF animals was even significantly attenuated compared to samples from sham-operated rats (Figs 2 and 3).

### *In vitro* effects of PETN on platelet adhesion and platelet VASP Ser^239^ phosphorylation

**Fig 4:**
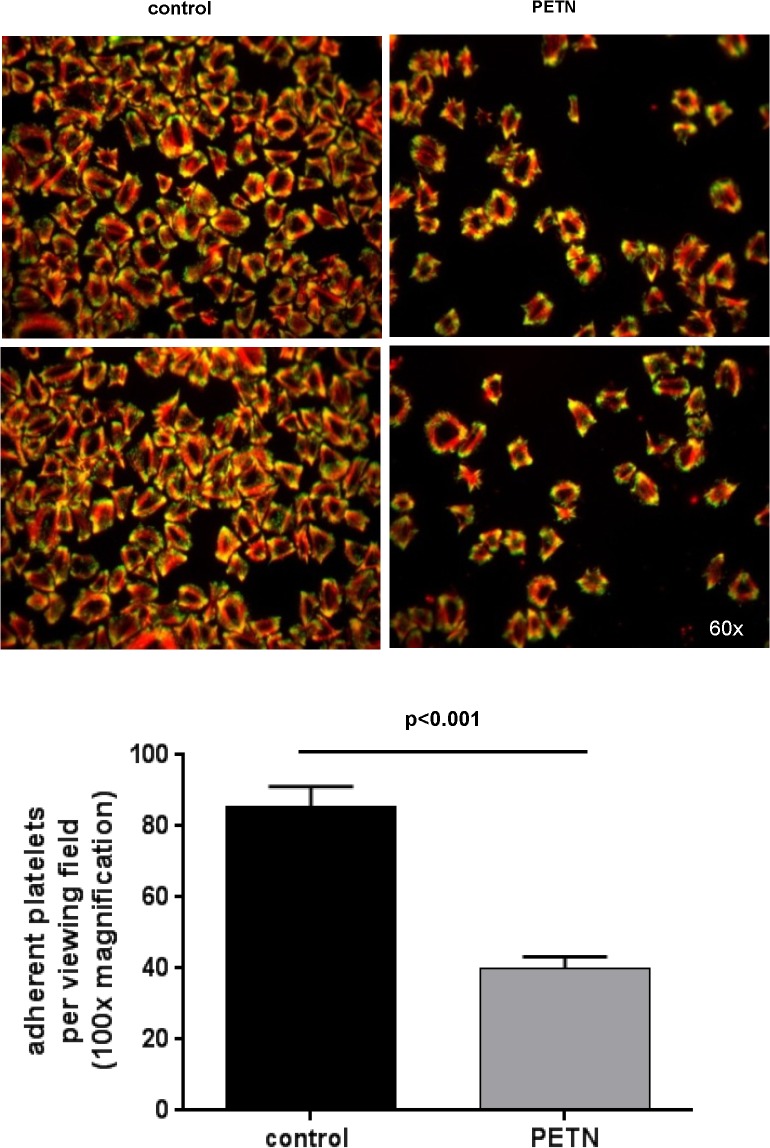
Impact of in vitro platelet incubation with PETN on platelet adhesion. (**A**) significantly reduced platelet adhesion to fibrinogen following PETN (5μg/mL) treatment compared to untreated control samples; (**B**) quantification of adhering platelets (p<0.001, n = 5).

**Fig 5:**
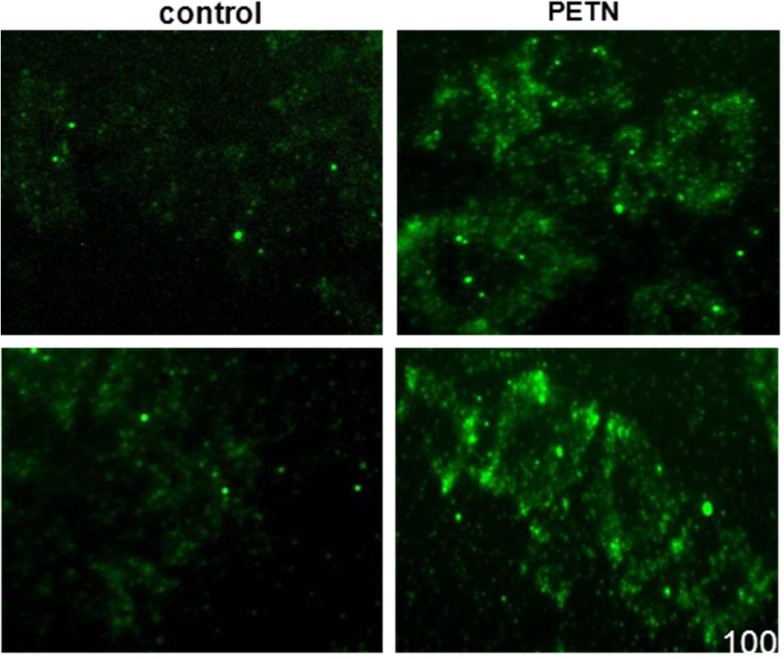
Impact of in vitro platelet incubation with PETN on VASP Ser^239^ phosphorylation. PETN pre-incubation of platelet samples significantly enhanced VASP Ser^**239**^ phosphorylation compared to unstimulated control samples; representative fluorescence images of 3 independently performed experiments.

The number of adhering platelets to fibrinogen coated glass slides was significantly reduced by 30 minute *in vitro* pre-incubation of PRP with PETN (5μg/mL) as compared to control samples which were pre-incubated with the solvent DMSO (number of adherent platelets: control: 85.6±5.5, PETN: 40±3.3; p<0.001, n = 5) (Fig 4). Moreover, staining for phosphorylated VASP at Ser^239^ revealed significantly enhanced antibody binding being a surrogate for significantly increased cGMP / cAMP availability in PETN treated platelets (Fig 5).

## Discussion

In this study we observed a significant attenuation of platelet activation and aggregation by chronic PETN administration in an animal model of heart failure. Increased platelet activation was normalized as shown by a significant reduction of fibrinogen binding compared to platelets from non-treated CHF animals (Fig 1). In line with that, increased platelet aggregability in the disease model was significantly attenuated following PETN treatment *in vivo* (Figs 2 and 3).

Pentaerythritol tetranitrate had been used as an anti-anginal drug since the late 1950s. However, in the following decades its application gradually abated due to lack of evidence concerning efficacy [22]. In the last years, PETN use increased after animal and human studies suggested beneficial endothelial effects without increased free radical production as observed upon GTN treatment [23, 24].

Indeed, PETN bears unique features within the group of nitrates. Besides its rather long duration of action of 10–12 hours, it is common consent that it does not cause nitrate tolerance, endothelial dysfunction or increased oxygen radical formation during long-term use [11]. Its predominant mechanism of action is the release of NO, the major target of which is soluble guanylyl cylcase (sGC). Activation of sGC leads to generation of cyclic guanosine monophosphate (cGMP) and subsequent cGMP-dependent protein kinase activation. In platelets, the activation of this signaling pathway finally prevents platelet activation, adhesion and aggregation [25]. The most recent PETN trials were conducted with CAD [18] and angina pectoris patients [19], two disease entities which are also associated with increased platelet activation [26]. Though the outcomes did not show an overall additional benefit compared to the control group, some improvements in exercise tolerance and microcirculation in subgroup analysis could still be observed. However, any possible impact of PETN on platelets was not investigated in these studies.

In addition to reduced platelet activation in heart failure in PETN treated animals, in the present study, we also demonstrate a direct effect of PETN on platelet adhesion *in vitro* (Fig 4). Moreover, we show that PETN treatment increases platelet VASP phosphorylation, a surrogate marker for enhanced platelet NO/cGMP signaling via soluble guanylyl cyclase activation (Fig 5) [25].

We cannot exclude that other protective mechanisms which are directly or indirectly influenced by PETN administration may play a role in our observed results. HO-1 for example, an enzyme which had been described to be a key player in the development of nitrate tolerance [27] was shown to be up-regulated in vascular tissue following PETN administration [17]. Interestingly, platelet rich thrombus formation in a ferric chloride carotid artery model was only significantly attenuated when HO-1 expression was upregulated, not however under basal conditions [28]. Moreover, reduced bioavailability of NO based on decreased endothelial NO generation and enhanced levels of ROS had been described to be one of the main reasons for the presence of increased platelet activation in heart failure [5]. Unlike other organic nitrates like GTN, ISMN or ISDN, which were shown to induce superoxide formation by activation of NAD(P)H oxidases, xanthine oxidases and mitochondrial redox reactions resulting in decreased NO production and NOS uncoupling, PETN was shown to exert quite contrary, more favourable effects [29]. Interestingly, NAD(P)H oxidases as well as mitochondria [30] were described to be relevant sources of ROS generation in platelets in general [31] and in heart failure patients [32]. Hence, PETN might also directly interact with platelet NAD(P)H oxidases and platelet mitochondrial redox reactions reducing ROS generation. However, measurement of platelet ROS production following PETN treatment was beyond the scope of this study and remains to be elucidated. Moreover, endothelial dysfunction and endothelial ROS production in heart failure are also important factors known to influence platelet activation [2]. Hence, beneficial effects of PETN on endothelial cells also affect platelet integrity in CHF.

Thus, the currently presented platelet effects of PETN might be multifactorial. However, sGC activation seems to play a major role as indicated by our *in vivo* and *in vitro* data. The beneficial role of sGC activation in reducing platelet activation had previously been observed in animal models of both CHF [33] and diabetes [34].

Clinically, there is no generally acknowledged prescription of antithrombotic or antiplatelet drugs for heart failure patients who do not present with atrial fibrillation, prosthetic valve replacement or left ventricular thrombus. This is mainly due to lack of evidence and due to the potential adverse effects like bleeding complications [35]. Significantly increased platelet activation is evident not only in severe heart failure or relying on the presence of atrial fibrillation but is also observed in patients with moderate heart failure [36] in sinus rhythm [37]. PETN prescription in heart failure is currently restricted to patients who are not eligible for coronary intervention but who still suffer from angina pectoris symptoms. According to our present data, administration of PETN which provides NO without inducing nitrate tolerance or endothelial dysfunction could, besides its beneficial anti-anginal and endothelial effects, also reduce thromboembolic events in heart failure by attenuating platelet activation without increasing the bleeding risk.

## Conclusion

Increased platelet activation in an animal model of congestive heart failure can be positively modulated by PETN. Hence, chronic NO supplementation using PETN reduces activation of circulating platelets as well as platelet reactivity following ADP stimulation in CHF rats. Thus, PETN may constitute a useful approach to help maintaining platelet integrity and thereby preventing thromboembolic complications in CHF.
